# Hepatoprotective Effect of Loquat Leaf Flavonoids in PM_2.5_-Induced Non-Alcoholic Fatty Liver Disease via Regulation of IRs-1/Akt and CYP2E1/JNK Pathways

**DOI:** 10.3390/ijms19103005

**Published:** 2018-10-01

**Authors:** Tunyu Jian, Xiaoqin Ding, Yuexian Wu, Bingru Ren, Weilin Li, Han Lv, Jian Chen

**Affiliations:** 1 Institute of Botany, Jiangsu Province and Chinese Academy of Sciences, Nanjing 210014, China; jiantunyu1986@163.com (T.J.); dingxiao_qin@126.com (X.D.); wuyuexian1993@163.com (Y.W.); bingruren@126.com (B.R.); lwlcnbg@cnbg.net (W.L.); 2 College of Forest, Nanjing Forestry University, Nanjing 210037, China

**Keywords:** PM_2.5_, Loquat Leaf, total flavonoids, non-alcoholic fatty liver disease, insulin resistance, oxidative stress, IRs/Akt, CYP2E1/JNK

## Abstract

Ambient air particulate matter (PM) represents a class of heterogeneous substances present in polluted air, which contains many harmful components. Exposure to ambient particulate matter in fine rages (PM_2.5_) is associated with non-alcoholic fatty liver disease (NAFLD). Loquat Leaf possesses pharmacological actions on NAFLD. As the main biological active ingredients, the potential therapeutic role of total flavonoids (TF) isolated from Loquat Leaf in PM_2.5_-induced NAFLD model remains unclear. The present study was designed to explore the hepatoprotective effect of TF in PM_2.5_-induced NAFLD mice with its related mechanisms of action. Mice were exposed to PM_2.5_ to induce NAFLD, and body weight, the ratio of liver to body weight, and blood lipids increased significantly compared with the control group. It was found that TF significantly reduced the above parameters in PM_2.5_-induced NAFLD mice. TF treatment alleviated oxidative stress by preventing the accumulation of oxidative product malondialdehyde (MDA) and by strengthening the anti-oxidative capacity of superoxide dismutase (SOD). TF was also found to reduce the alanine aminotransferase (ALT) and aspartate aminotransferase (AST) activity in the PM_2.5_ group. In addition, TF repaired the PM_2.5_-induced decline of insulin receptor substrate-1 (IRs-1) and protein kinase B (Akt) phosphorylation. Meanwhile, the data showed TF suppressed the expression of cytochrome P450 2E1(CYP2E1) and the phosphorylation of c-jun N-terminal kinase (JNK) in PM_2.5_-induced NAFLD. Taken together, these findings show that TF alleviate PM_2.5_-induced NAFLD via regulation of IRs-1/Akt and CYP2E1/JNK pathways, which may have potential for further development as novel therapeutic agents for NAFLD.

## 1. Introduction

Ambient air particulate matter (PM) represents a class of heterogeneous substances present in polluted air, especially the ambient fine particulate matter (aerodynamic diameter < 2.5 μm, PM_2.5_), which has been associated with the increasing prevalence of many diseases, such as pulmonary, cardiovascular, and hepatic diseases and metabolic syndrome [1,2,3,4]. PM_2.5_ is a complex mixture of particles and gases which originates from automobile exhaust, secondary sulfate, smoke, soil, and aged sea salt [5]. The chemical and biological elements of PM_2.5_ include metals, salts, volatile organic compounds, hydrocarbons, and even endotoxins [6,7]. These PM_2.5_ particles not only can deposit in alveolar regions of the lung, but also can penetrate into the distal airway units and enter into the circulatory system with diminishing sizes, bringing health risks [8].

Non-alcoholic fatty liver disease (NAFLD) is a chronic liver disease characterized by hepatic steatosis, and it is also regarded as a manifestation of metabolic syndrome in the liver. It refers to a wide spectrum of liver disease developing progressively from simple steatosis to non-alcoholic steatohepatitis (NASH), fibrosis, and even cirrhosis [9,10]. It has been revealed that the inhalation of PM_2.5_ has direct and profound effects on the liver, which is the principle detoxification and metabolic organ. Multiple studies have shown that PM_2.5_ exposure induces integrated lipid accumulation, oxidative stress, insulin resistance, and inflammatory responses in the liver, leading to NAFLD in animal models [2,3,11,12,13].

Although the detailed pathogenesis of NAFLD has not been resolved completely, steatosis and oxidative stress are believed to play important roles in the progression of NAFLD [14]. As a member of metabolic disorders, hepatic lipid accumulation is strongly associated with insulin resistance (IR) [15]. Insulin receptor substrate-1 (IRs-1) is responsible for the transduction of insulin signaling, and IRs-1 phosphorylation at Tyr leads to insulin pathway activation [16]. Protein kinase B (Akt) is also a crucial component of insulin signaling cascade. In the NAFLD model, IR is accompanied with reduced phosphorylation of Akt, and activated Akt could ameliorate hepatic steatosis [17]. Moreover, as a member of the mixed-function oxidase system, cytochrome P450 2E1 (CYP2E1) is responsible for oxidizing a variety of endogenous and exogenous substances (including fatty acids, ethanol, most organic solvents, and even environmental contaminants) [18,19]. In NAFLD, overexpression of CYP2E1 accelerates the production of reactive oxygen species (ROS) leading to oxidative stress. This effect is associated with sustained activation of C-jun N-terminal kinase (JNK) signaling cascades which progressed hepatocellular injury and microvesicular steatosis [19]. Recent reports indicated that PM_2.5_ exposure inhibited the IRs-1/Akt signal pathway, leading to IR, which impaired glucose metabolism in the liver [13]. However, the relationship between IR, oxidative stress, and PM_2.5_-induced NAFLD needs to be further investigated.

As a traditional Chinese medicine, Loquat Leaf (*Eriobotrya japonica* (Thunb.) Lindl (Rosaceae)) has been widely used in the treatment of respiratory diseases for centuries, such as pharyngolaryngitis, bronchitis, chronic bronchitis cough, and chronic obstructive pulmonary disease (COPD) [20]. Extracts of Loquat Leaf also have shown other bioactivities, especially the effect on metabolic disorders, including anti-diabetic, hypolipidemic, and anti-obesity activities [21,22,23]. As a hepatic manifestation of the metabolic syndrome, Loquat Leaf extracts also have therapy effect on NAFLD [19]. Phytochemical studies indicate that flavonoids are a member of the main chemical components of Loquat Leaf, and most of them contain quercetin and kaemferol as the parent nucleus, such as rutin (quercetin-3-*O*-rutinoside), kaemferol-3-*O*-glucoside, and so on [24]. These flavonoids are known as powerful polyphenols and antioxidants, and they also have potent effects on metabolic syndrome, such as hyperglycemia, hyperlipidemia [24,25], and anti-NAFLD activities [26]. In our previous study, we demonstrated that total flavonoids (TF) isolated from Loquat Leaf displayed a beneficial effect on glucose metabolism disorder [24]. However, to the best of our knowledge, the efficacy and mechanism of TF isolated from Loquat Leaf in PM_2.5_ induced NAFLD mouse model remain unclear.

In this study, results showed that TF isolated from Loquat Leaf could improve hepatic steatosis and oxidative stress in a PM_2.5_-induced NAFLD mice model. Moreover, its related mechanism was also demonstrated. This study may provide a reference for the development of anti-NAFLD drug discovery from natural compounds.

## 2. Results

### 2.1. Analysis of Chemical Constituents of TF by HPLC-QTOF/MS

**Figure 1:**
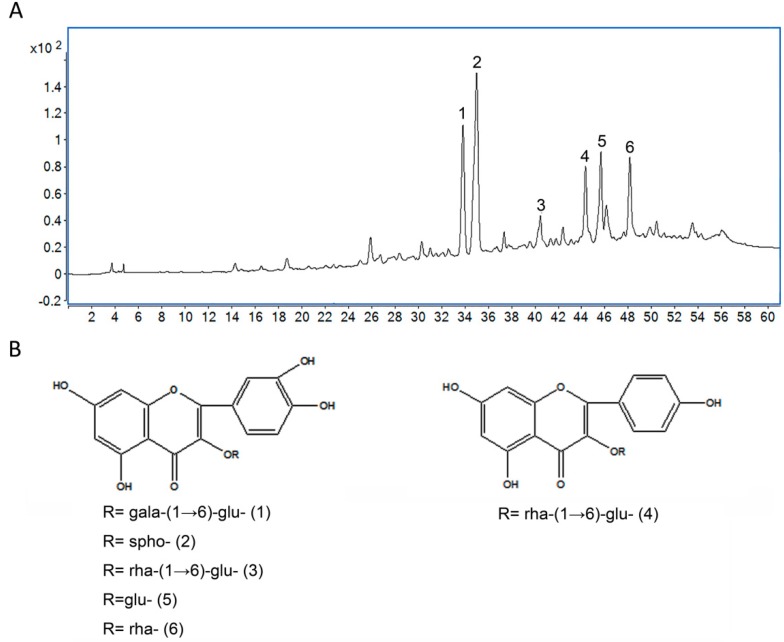
The chromatogram of total flavonoids (TF) analysis by HPLC-QTOF/MS (**A**) and the chemical structures of the six main flavonoids in TF (**B**).

In order to understand approximately the bioactive components in TF, the detection was conducted by using HPLC-QTOF/MS. The total ion current chromatogram of TF in positive mode was shown in Figure 1A. According to elution order, the main peaks at R_t_ = 33.87, 35.13, 41.06, 44.32, 45.68, and 48.14 were flavonoids, the amount of six represented flavonoids in TF accounting for 83.2 ± 2.7%. Their chemical structures were presented in Figure 1B, which were identified in our previous study [24].

### 2.2. Effect of TF on the Body and Liver Weight in Mice Exposed to PM_2.5_

**Figure 2:**
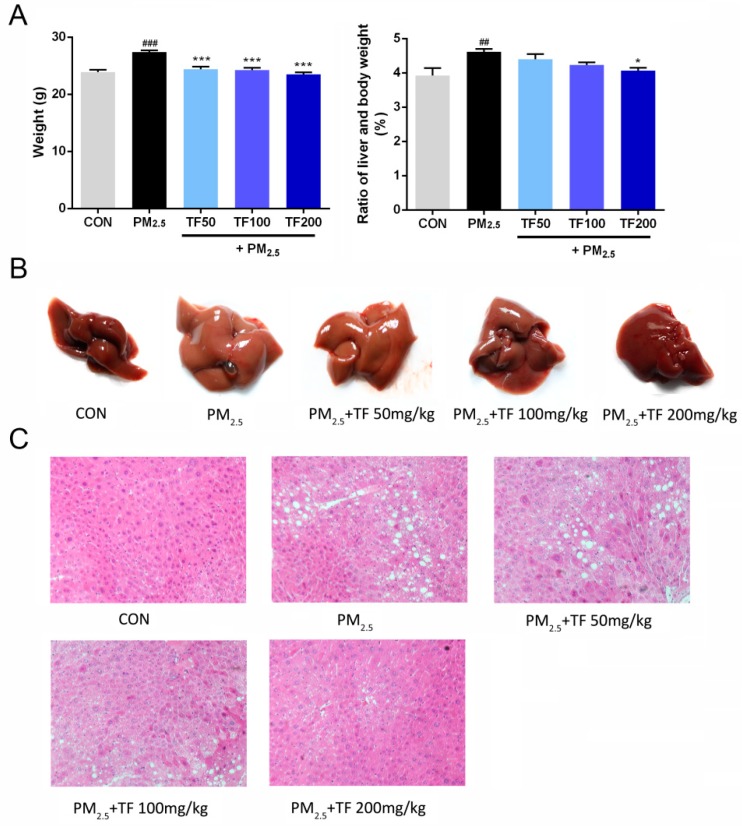
Body weight and ratios of liver to body weight (%) (**A**); appearance of the liver (**B**); histological analysis of liver tissues (H&E, 200× magnification) (**C**). Data are presented as mean ± SE (*n* = 10). ^##^ *p* < 0.01, ^###^ *p* < 0.001 vs. the CON group; * *p* < 0.05, *** *p* < 0.001 vs. the PM_2.5_ group.

As shown in Figure 2A, after 35 days of exposure to PM_2.5_, mice in the PM_2.5_ group exhibited an obvious increase in body weight and ratios of liver to body weight (%) compared with the control group (*p* < 0.001 and *p* < 0.01, respectively). Compared with PM_2.5_ group, the body weight of PM_2.5_-TF (50, 100 and 200 mg/kg) groups had markedly decreased (*p* < 0.001). It was also shown that after treatment with a high dose of TF (200 mg/kg), the ratio of liver to body weight decreased significantly (*p* < 0.05). Treatment with TF inhibited the progression of weight gain in the body and liver tissue.

### 2.3. Histopathological Examinations

In the control group, the livers were deep red, glossy, and resilient. Meanwhile, in the PM_2.5_ exposure group, the livers were enlarged, lost luster, and had yellow necrosis foci on the surface. As shown in Figure 2B, liver appearance in the TF treated mouse was improved in a dose-dependent manner.

H&E staining was used to evaluate liver histology (Figure 2C). The results indicated that, in the normal mice, liver tissues exhibited well-arranged normal liver lobular structure, and with neither fatty degeneration nor inflammatory signs. However, exposure to PM_2.5_ could markedly increase the disorder of the hepatic lobule structures, and lipid droplets and ballooning degeneration were observed in the most of liver cells. As revealed in Figure 2C, after treatment with TF extracted from Loquat Leaf, the mitigated microvesicular fatty and histopathological changes in liver tissue were improved in a dose-dependent manner, and the volume and number of lipid droplets markedly decreased compared to PM_2.5_ exposure animals. TF treatment significantly ameliorated the steatosis and hepatocyte swelling, especially in the treatment of TF at a high dose of 200 mg/kg, and the morphology of the liver lobular structure recovered to nearly normal status.

### 2.4. Improvement of TF on Lipid Metabolism

**Figure 3:**
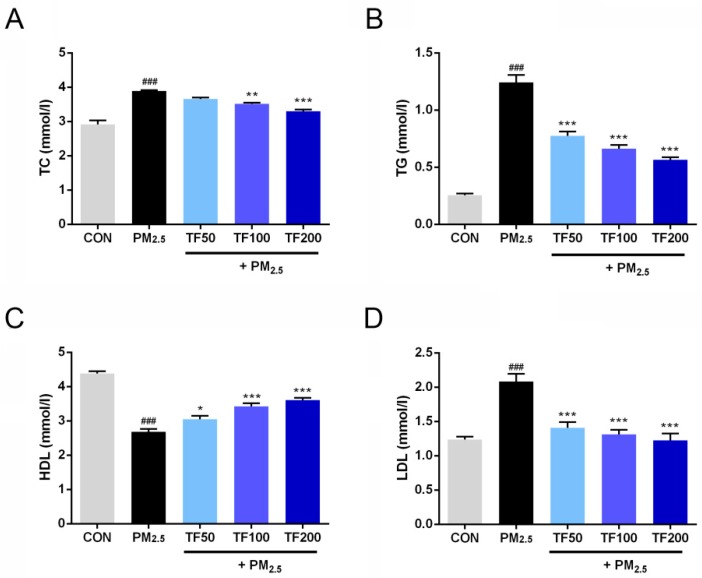
Effects of TF in PM_2.5_ exposure induced NAFLD on plasma total cholesterol (TC) (**A**), triglycerides (TG) (**B**), high-density lipoprotein cholesterol (HDL) (**C**), and low-density lipoprotein cholesterol (LDL) (**D**) in the following groups: Control group (CON), PM_2.5_ exposure group, PM_2.5_ + TF 50 mg/kg group, PM_2.5_ + TF 100 mg/kg group and PM_2.5_ + TF 200 mg/kg group. All the results were presented as mean ± SE (*n* = 10). ^###^ *p* < 0.001 vs. the CON group; * *p* < 0.05, ** *p* < 0.01, *** *p* < 0.001 vs. the PM_2.5_ group.

To assess the impacts of PM_2.5_ exposure on lipid metabolism, relevant parameters were detected. At the end of experimental period, the serum total cholesterol (TC), triglycerides (TG), and low-density lipoprotein cholesterol (LDL) level showed significant increase in mice under PM_2.5_ exposure compared to the control group (*p* < 0.001; Figure 3A,B,D). On the contrary, the serum high-density lipoprotein cholesterol (HDL) significantly decreased (*p* < 0.001; Figure 3C). It was observed that administration of TF (100 and 200 mg/kg) markedly decreased the levels of TC, TG, and LDL, and increased the level of HDL in PM_2.5_ + TF groups (*p* < 0.01; Figure 3A–D). Compared with the PM_2.5_ exposure group, TG and LDL level significantly decreased in PM_2.5_ + TF 50 mg/kg group (*p* < 0.001; Figure 3B,D). Furthermore, HDL was increased (*p* < 0.05; Figure 3C), but no significant change of the level of TC was observed in PM_2.5_ + TF 50 mg/kg group (*p* > 0.05; Figure 3A).

### 2.5. Reduced Oxidative Stress and Liver Injury in PM_2.5_-Induced NAFLD Mice

**Figure 4:**
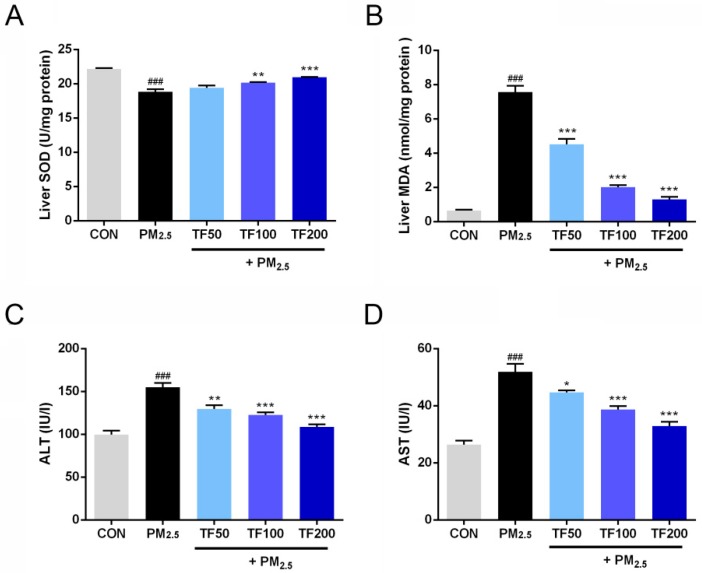
Effects of TF administration on oxidative stress and liver injury in PM_2.5_ induced NAFLD. The levels of superoxide dismutase (SOD) (**A**), malondialdehyde (MDA) (**B**) in the liver, alanine aminotransferase (ALT) (**C**) and aspartate aminotransferase (AST) (**D**) in the serum were measured in the following groups: control group (CON), PM_2.5_ exposure group, PM_2.5_ + TF 50 mg/kg group, PM_2.5_ + TF 100 mg/kg group and PM_2.5_ + TF 200 mg/kg group. All the results were presented as mean ± SE (*n* = 10). ^###^ *p* < 0.001 vs. the CON group; * *p* < 0.05, ** *p* < 0.01, *** *p* < 0.001 vs. the PM_2.5_ group.

As displayed in Figure 4A,B, compared with control group, liver tissue from PM_2.5_ exposure group displayed less abundant SOD and excessive accumulation of MDA, indicating the imbalance between anti-oxidative capacity and oxidative stress in PM_2.5_-induced NAFLD liver. As extraneous supplementation of anti-oxidant, TF administration partially rectified this imbalance by increasing SOD content and reducing MDA generation (Figure 4A,B). As important indicators of liver damage, ALT and AST levels were detected to monitor liver function. Compared with the control group, levels of ALT and AST were significantly increased in the PM_2.5_ exposure group (*p* < 0.001; Figure 4C,D). TF treatment proved to be advantageous for reducing ALT and AST levels in PM_2.5_ induced NAFLD mice (*p* < 0.05; Figure 4C,D). These findings suggest that TF treatment can protect against liver injury induced by PM_2.5_ exposure.

### 2.6. TF Changes Pathways Involved in Insulin Resistance and Oxidative Stress

**Figure 5:**
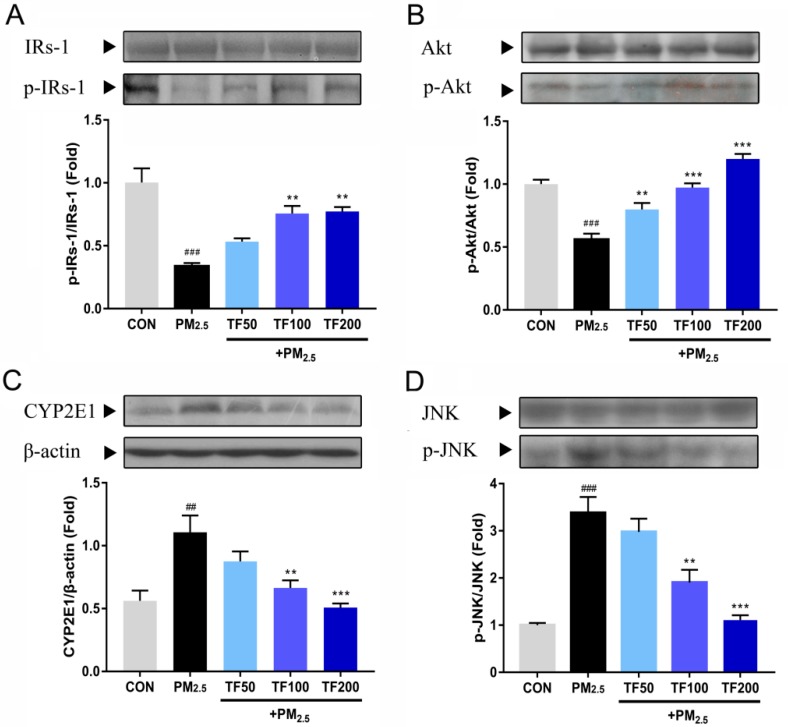
Effects of TF on insulin receptor substrate-1 (IRs-1), protein kinase B (Akt), cytochrome P450 2E1 (CYP2E1), and c-jun N-terminal kinase (JNK) in the liver tissue of PM_2.5_ exposure induced NAFLD mice. (**A**) IRs-1, p-IRs-1, (**B**) Akt, p-Akt, (**C**) CYP2E1, (**D**) JNK, and p-JNK expression were probed by western blotting. All the results were presented as mean ± SE (*n* = 3). ^##^ *p* < 0.01, ^###^ *p* < 0.001 vs. the CON group; ** *p* <0.01, *** *p* < 0.001 vs. the PM_2.5_ group.

To further explore the hepatoprotective mechanism of TF, we tested the expression of IRs-1, Akt, CYP2E1, and JNK in liver tissues, which are involved in insulin resistance and oxidative stress. As showed in Figure 5A,B, the phosphorylation of IRs-1 and Akt were significantly attenuated in the liver of mice after PM_2.5_ exposure (*p* < 0.001); meanwhile, CYP2E1 and the phosphorylation of JNK dramatically increased (*p* < 0.01, Figure 5C,D). TF administration repaired PM_2.5_ exposure induced inhibition of IRs-1 and Akt phosphorylation in a dose-dependent manner (*p* < 0.01, Figure 5A,B). In addition, TF treatment brought a significant decrease of CYP2E1 expression and JNK activation at a high dose (100 and 200 mg/kg, Figure 5C,D).

## 3. Discussion

**Figure 6:**
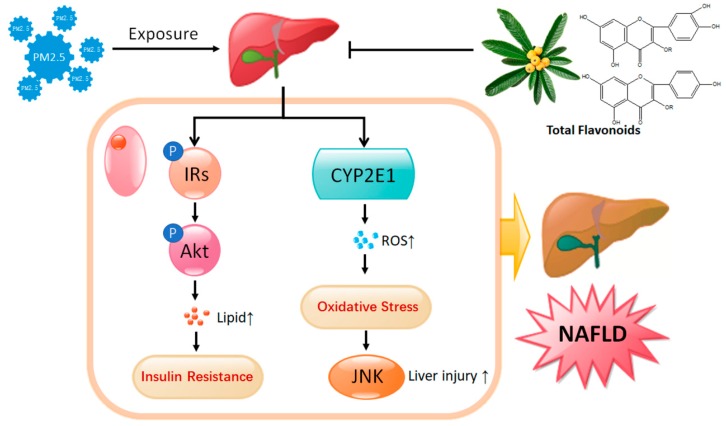
A schematic illustration of how TF extracted from Loquat Leaf interfered with IR and oxidative stress in PM_2.5_-induced NAFLD. “↑” indicates increase.

In this study, we demonstrated that PM_2.5_ exposure for 35 days caused significant lipid metabolism disorder, liver damage, and oxidative stress. We further found that exposure to PM_2.5_ can inhibit IRs/Akt signaling, which participated in IR promotion. On the other hand, it can increase the expression of CYP2E1 and p-JNK, which is related to the promotion of oxidative stress. Both the IR and oxidative stress are involved in the pathogenesis of NAFLD. Administration of Total flavonoids (TF) isolated from Loquat Leaf directly reduced lipid accumulation by up-regulating p-IRs-1 and p-Akt protein content. TF treatment can also inhibit oxidative stress by down-regulating the expression of CYP2E1 and p-JNK (Figure 6).

With the growing population in our society and rapid development of the social economy, environmental pollution has posed a serious threat to human health and human society, and PM_2.5_ is believed to be the primary cause of air pollution. It has been widely reported that PM_2.5_ can enter the respiratory system and diffuse into the blood circulation, inducing a series of metabolic syndromes, such as diabetes [27], hepatic steatosis [12], NASH-like phenotype, and hepatic glucose metabolism damage [3]. As a hepatic manifestation of the metabolic syndrome, NAFLD shares several pathological characteristics with insulin resistance (IR) and excessive oxidative stress [28]. Recent studies showed that exposure to PM_2.5_ decreased glucose consumption or tolerance by inhibiting the insulin signaling pathway and triggered reactive oxygen species (ROS) generation which induced oxidative stress in hepatocytes and liver tissue of mice [13,29]. However, there is still no definitive and effective treatment for NAFLD induced by PM_2.5_ exposure as its pathology remains to be elucidated.

Loquat Leaf has been widely used in the treatment of respiratory diseases since ancient time, which has a strong effect on metabolic disorders and oxidative stress [23]. In our previous study, Loquat Leaf exhibited a therapeutic effect on high-fat-diet induced NAFLD by regulating lipid accumulation and oxidative stress [19]. Flavonoids (about 3% in total extract) containing quercetin and kaemferol as the parent nucleus are main active components in Loquat Leaf [24,30]. These flavonoids not only have benefits on glucose metabolism, lipid metabolism, and NAFLD, but also exhibit anti-oxidative activity [23,24,25,26]. In this study, after enrichment and purification, six flavonoids were identified, and the content of TF in Loquat Leaf is 83.2 ± 2.7%. We further investigated the effect and mechanism of TF on PM_2.5_ exposure-induced NAFLD.

Lipid accumulation in the liver is regarded as the “first hit” in the pathogenesis of NAFLD [14]. Change of liver appearance and widespread of lipid vacuoles and ballooning degeneration inside the lobule structures proved that the NAFLD model was established successfully by PM_2.5_ exposure. Several studies have suggested that lipid metabolism is regulated mainly by the insulin signaling pathway [31,32], and hepatic lipid accumulation is strongly associated with insulin resistance (IR) [15]. Insulin receptor substrate-1 (IRs-1) as a protein phosphorylated by insulin receptor tyrosine kinase, is responsible for the transduction of insulin signaling. Impaired responsiveness to insulin leads to IR, which typically occurs at the level of IRs-1, has been observed in metabolic disorders, including type 2 diabetes and NAFLD [33,34]. Restoration of IRs-1 is essential for insulin signaling, which might be beneficial for attenuating NAFLD [35]. Along with IRs-1, Protein kinase B (Akt) is the other crucial component of insulin signaling cascade. After binding of insulin to the insulin receptor, IRs is phosphorylated at Tyr and subsequently triggers the activation of Akt [36]. IR was accompanied with reduced phosphorylation of Akt in NAFLD, and maintenance of an appropriate Akt level could ameliorate hepatic steatosis [17]. Therefore, the modulation of IRs-1/Akt may be an effective way to improve insulin sensitivity, which is involved in lipid metabolism of NAFLD. A recent study revealed that PM_2.5_ exposure increased IRs phosphorylation at Ser^307^, but reduced Akt phosphorylation at Ser^473^, which resulted in insulin resistance in liver [13]. In this study, in the PM_2.5_-induced NAFLD model, TF regulating the IRs-1/Akt pathway produced pharmaceutical effects on excessive lipids accumulation (including TC, TG, HDL, and LDL), which was associated with IR. This might be part of the mechanism underlying PM_2.5_-induced NAFLD improvement by TF.

Oxidative stress was considered as the “second hit” in the pathogenesis of NAFLD. As a member of the oxidoreductase cytochrome family, Cytochrome P450 2E1 (CYP2E1) is responsible for oxidizing a variety of small molecule substrates including fatty acids, organic solvents, and environmental contaminants [18,19]. In NAFLD, the capacity of CYP2E1 to generate ROS is critical to induced oxidative stress [37]. As an essential member of enzymatic antioxidant defense systems, SOD is responsible for protecting against the toxic effects induced by ROS. In addition, MDA is a kind of oxidative product, which contributes to apoptosis and DNA damage in NAFLD by producing ROS [19]. Many studies have shown that increasing expression of CYP2E1 makes a significant contribution to oxidative stress in NAFLD livers by increasing MDA level and decreasing SOD level. Absence or blocking of CYP2E1 may exert beneficial effects on NAFLD by inhibiting oxidative stress [19,38,39,40,41]. Our data suggested that PM_2.5_ exposure induced the over expression of CYP2E1, and the anti-oxidative stress effect of TF is mediated by SOD and MDA via inhibiting CYP2E1 expression. In addition, in the NAFLD condition, oxidative stress induced by CYP2E1 activation triggered the activation of JNK signaling cascades. Higher levels of CYP2E1 and phospho-JNK were observed in NAFLD, and after the CYP2E1 deletion, JNK phosphorylation also disappeared. JNK is a potent cell death signaling pathway which regulates hepatocellular injury [40,41,42]. ALT and AST are important indicators of liver injury, both of which showed an increase in mice under PM_2.5_ exposure. However, after treatment with TF, liver injury parameters were recovered. Taken together, we speculate that CYP2E1/JNK is an essential therapeutic target for PM_2.5_-induced NAFLD and TF might be a natural product which could alleviate PM_2.5_-NAFLD associated hepatocyte damage via inhibiting CYP2E1/JNK.

## 4. Materials and Methods

### 4.1. Chemicals, Reagents, and Antibodies

The solvents and chemicals used for the mobile phase in HPLC-QTOF/MS analysis were purchased as follows: Methanol (LC-MS grade) was from Fisher scientific (Schwerte, Germany) and formic acid (HPLC grade) was from Acros Organics (Geel, Belgium). Water (LC-MS grade) was freshly generated by a Milli-Q reagent water system (Millipore, Bedford, MA, USA). Other solvents and reagents were obtained from Merck (Darmstadt, Germany) in analytical grade purity.

Protein extraction kit and BCA protein assay kit were purchased from KeyGen Biotechnology (Nanjing, China). The commercial kits for quantifying total cholesterol (TC), triglycerides (TG), high-density lipoprotein cholesterol (HDL), low-density lipoprotein cholesterol (LDL), alanine aminotransferase (ALT), aspartate aminotransferase (AST), superoxide dismutase (SOD), and malonaldehyde (MDA) were purchased from Nanjing Jiancheng Bioengineering Institute (Nanjing, China).

The following primary antibodies were used for the Western blotting (WB) assay: CYP2E1 (ab28146, 1:1000) and β-actin (ab8227, 1:2000) were purchased from Abcam (Cambridge, MA, USA). JNK (#9252, 1:1000), p-JNK (#9251, 1:1000), IRs-1 (#2382, 1:1000), p-IRS-1 (Tyr895) (#3070, 1:1000), Akt (#4691, 1:1000), p-Akt (Ser473) (#9271, 1:1000) were obtained from Cell Signaling Technology (Danvers, MA, USA). Anti-rabbit IgG, HRP-linked Antibody (#7074, 1:3000) and Anti-mouse IgG, HRP-linked Antibody (#7076, 1:3000) were used as secondary antibody, which were purchased from Cell Signaling Technology (Danvers, MA, USA).

### 4.2. Preparation and Analysis of TF from Loquat Leaf

The Loquat Leaves were collected from Suzhou, Jiangsu, China (120.296235 S; 31.086002 W). The voucher specimen (No. 328636) was deposited at the Herbarium of the Institute of Botany, Jiangsu Province and Chinese Academy of Sciences.

TF from Loquat Leaf was prepared according to the previously reported method [24]. TF was dissolved in 50% methanol at a concentration of 2 mg/mL and filtered through a 0.45-μm PVD filter. For the chemical analysis of TF obtained, a 6530 Accurate-Mass LC-QTOF-MS device (Agilent Technologies, Santa Clara, CA, USA) was used for all measurements. Chromatographic separation was carried out using an Agilent 1260 Infinity LC system equipped with an Agilent ZORBAX SB-C18 column (1.8 µm, 4.6 × 100 mm; Waldbronn, Germany). The column temperature was held at 30 °C. The injection volume was 10 μL. Methanol (A) and 0.1% formic acid (B) were used as the mobile phase under gradient condition (0–60 min, 15–55% A).

The HPLC system was connected to a QTOF-MS equipped with an electrospray ion source ESI, operating in the positive ion mode with the following operation parameters: capillary voltage, 4 kV; nebulizer pressure, 50 psi; drying gas, 10 L/min; gas temperature, 350 °C. The full-scan MS spectrum was acquired over a mass range of 100–1000 m/z at 4 GHz high resolution. The measurement and post-run analysis were controlled by the software MassHunter Acquisition B0.05.0 (Agilent Technologies, Palo Alto, CA, USA).

### 4.3. Animals

All animal experiments for the study followed the Guide for the Care and Use of Laboratory Animals and were approved by the Animal Ethics Committee of China Pharmaceutical University (certificate number: SYXK2016-0011, approval date: 27 January 2016 to 26 January 2021), meanwhile, appropriate steps were taken to reduce pain or discomfort of the mice. Male ICR (Institute of Cancer Research) mice 6–8 weeks of age and weight 17–25 g, were purchased from Sino-British SIPPR/BK Lab Animal Co., Ltd. (Shanghai, China; certificate no. SCXK2013-0016). The animals were fed in a controlled temperature (23 ± 2 °C) and humidity (60 ± 5%) room with 12:12 light/dark cycle in Laboratory Animal Center of China Pharmaceutical University (Nanjing, China). All the animals had free access to diet and tap water.

### 4.4. Exposure Protocol

All PM_2.5_ samples used during this study were collected from Nanjing, China using a PM_2.5_ high volume sampler system (Staplex PM_2.5_, SSI, New York, NY, USA) with glass fiber filters (20.3 × 25.4 cm) as described previously [43,44]. PM_2.5_-containing filter membranes were sonicated with ultrapure water to obtain the PM_2.5_ suspension. The collected PM_2.5_ suspension was concentrated and dried in a vacuum freeze-drying machine, and then packed in clean aluminum foil and stored at −20 °C until they were used.

The mice were acclimatized for 1 week, and were randomly divided into 5 groups of 10 animals each: Control, PM_2.5_, PM_2.5_ + TF 50 mg/kg, PM_2.5_ + TF 100 mg/kg and PM_2.5_ + TF 200 mg/kg. Then, mice were anesthetized by intraperitoneal injection of 5% chloral hydrate, while the PM_2.5_-treated groups were intratracheally instillized PM_2.5_ particles at the dosage of 40 mg/kg every 7 days for 35 days. The control group was treated with an equal volume normal saline. For 35 consecutive days, the PM_2.5_ + TF groups received different dosage of TF orally daily, while the mice in the control and PM_2.5_ groups were administered equivalent volumes of saline.

### 4.5. Blood and Tissue Collection

At the end of 35 days of PM_2.5_ exposure, after 12 h fasting, all animals were sacrificed under chloral hydrate anesthesia. Their whole blood samples were collected from the abdominal vein and were immediately placed into ice-chilled tubes for 20 min. The blood samples were separated at 3000 rpm for 10 min at 4 °C to obtain serum, which was then stored at −80 °C for further biochemical analysis. Liver tissues were carefully dissected, photographed, weighed, and then frozen in liquid nitrogen and stored at −80 °C prior to analysis.

### 4.6. Biochemical Parameters Analysis

A Molecular Devices Spectra Max Plus automatic plate reader (Molecular Device, Sunnyvale, CA, USA) was used to determine the serum levels of TC, TG, HDL, LDL, ALT, and AST by commercial kits according to the manufacturer’s instructions. To determine SOD and MDA levels, the frozen liver tissues were homogenized in phosphate buffer solution (PBS). SOD and MDA contents in the supernatants were detected using commercial kits. All experiments were analyzed in triplicate, and the average values are presented.

### 4.7. Liver Histology Assessment

Liver samples from Control- and PM_2.5_-treated mouse were fixed in 4% paraformaldehyde overnight and then embedded in paraffin and sectioned (4 μm). The liver sections were stained with hematoxylin & eosin (H&E) according to standard instruction [45]. Each section was examined by a specialist who had no knowledge about the sample information, using an Axio Zoom.V16 Zoom Microscopefrom Carl Zeiss (Carl Zeiss, Oberkochen, Germany) at 200× magnification.

### 4.8. Western Blotting

Fresh liver tissues were homogenized and lysed in ice-cold buffer by tissue homogenizer (FSH-2A, LanKai, Nanjing, China), and then tissue lysate samples were centrifuged at 12,000 rpm at 4 °C for 5 min, and protein concentrations were measured by a BCA protein assay. Denatured proteins (20 μg) were separated on the SDS-polyacrylamide gel electrophoresis (SDS-PAGE) and transferred to a 0.45 μm PVDF membrane (Millipore, Bedford, MA, USA). The membrane was washed in Tris-buffered saline (TBS) and blocked with 5% non-fat milk at room temperature for 2 h. The membranes were incubated overnight at 4 °C with primary antibody, and then incubated with secondary antibody at room temperature for 2 h. Membrane-bound antibodies were detected by an enhanced chemiluminescence (Santa Cruz Biotechnology, Santa Cruz, CA, USA). The luminescence signal was captured with a Fuji medical X-ray film (Fujifilm, Tokyo, Japan). β-Actin was used as a loading control for total protein content.

### 4.9. Statistical Analysis

All of the data were obtained from three separate experiments and expressed as the mean ± standard error (SE) from 10 mice. The statistical significance was evaluated using one-way ANOVA with a post-hoc test by GraphPad Prism 7.0 (GraphPad Software, Inc., San Diego, CA, USA). A value of *p* < 0.05 indicated statistical significance.

## 5. Conclusions

In summary, we provide evidence that administration of total flavonoids (TF) isolated from Loquat Leaf significantly improve the metabolic parameters and oxidative stress in NAFLD mice induced by PM_2.5_ exposure. The mechanisms may be attributed to IRs/Akt activation and CYP2E1/JNK inhibition. TF extract from Loquat Leaf could be a nutraceutical to prevent PM_2.5_-associated obesity and NAFLD, however, this requires further investigation.

## Abbreviations

PM_2.5_	Ambient fine particulate matter (aerodynamic diameter < 2.5 μm)
NAFLD	Non-alcoholic fatty liver disease
TF	Total flavonoids
MDA	Malondialdehyde
SOD	Superoxide dismutase
ALT	Alanine aminotransferase
AST	Aspartate aminotransferase
IRs-1	Insulin receptor substrate-1
Akt	Protein kinase B
CYP2E1	Cytochrome P450 2E1
JNK	c-Jun N-terminal kinase
NASH	Non-alcoholic steatohepatitis
IR	Insulin resistance
ROS	Reactive oxygen species
COPD	Chronic obstructive pulmonary disease
TC	Total cholesterol
TG	Triglycerides
HDL	High-density lipoprotein cholesterol
LDL	Low-density lipoprotein cholesterol
PBS	Phosphate buffer solution
H&E	Hematoxylin & eosin
SDS-PAGE	SDS-polyacrylamide gel electrophoresis
TBS	Tris-buffered saline
SE	Standard error
